# Polymorphisms associated with everolimus pharmacokinetics, toxicity and survival in metastatic breast cancer

**DOI:** 10.1371/journal.pone.0180192

**Published:** 2017-07-20

**Authors:** Tomas Pascual, María Apellániz-Ruiz, Cristina Pernaut, Cecilia Cueto-Felgueroso, Pablo Villalba, Carlos Álvarez, Luis Manso, Lucia Inglada-Pérez, Mercedes Robledo, Cristina Rodríguez-Antona, Eva Ciruelos

**Affiliations:** 1 Medical Oncology Department, 12 de Octubre University Hospital, Madrid, Spain; 2 Hereditary Endocrine Cancer Group, Human Cancer Genetics Programme, Spanish National Cancer Research Centre (CNIO), Madrid, Spain; 3 Biochemistry Department, 12 de Octubre University Hospital, Madrid, Spain; 4 Centro de Investigación Biomédica en Red de Enfermedades Raras (CIBERER), Madrid, Spain; Southern Illinois University School of Medicine, UNITED STATES

## Abstract

**Purpose:**

Metastatic breast cancer (MBC) progressing after endocrine therapy frequently activates PI3K/AKT/mTOR pathway. The BOLERO-2 trial showed that everolimus-exemestane achieves increased progression free survival (PFS) compared with exemestane. However, there is great inter-patient variability in toxicity and response to exemestane-everolimus treatment. The objective of this study was to perform an exploratory study analyzing the implication of single nucleotide polymorphisms (SNPs) on outcomes from this treatment through a pharmacogenetic analysis.

**Patients and methods:**

Blood was collected from 90 postmenopausal women with hormone receptor-positive, HER2-negative MBC treated with exemestane-everolimus following progression after prior treatment with a non-steroidal aromatase inhibitor. Everolimus pharmacokinetics was measured in 37 patients. Twelve SNPs in genes involved in everolimus pharmacokinetics and pharmacodynamics were genotyped and associations assessed with drug plasma levels, clinically relevant toxicities (non-infectious pneumonitis, mucositis, hyperglycemia and hematological toxicities), dose reductions or treatment suspensions due to toxicity, progression free survival (PFS) and overall survival.

**Results:**

We found that *CYP3A4* rs35599367 variant (CYP3A4*22 allele) carriers had higher everolimus blood concentration compared to wild type patients (P = 0.019). *ABCB1* rs1045642 was associated with risk of mucositis (P = 0.031), while *PIK3R1* rs10515074 and *RAPTOR* rs9906827 were associated with hyperglycemia and non-infectious pneumonitis (P = 0.016 and 0.024, respectively). Furthermore, *RAPTOR* rs9906827 was associated with PFS (P = 0.006).

**Conclusions:**

CYP3A4*22 allele influenced plasma concentration of everolimus and several SNPs in PI3K/AKT/mTOR pathway genes were associated with treatment toxicities and prognosis. These results require replication, but suggest that germline variation could influence everolimus outcomes in MBC.

## Introduction

Breast cancer is a life-threatening disease and is the second leading cause of cancer death among women. It has been estimated that in 2017 there will be 255,180 newly diagnosed breast cancer cases in the US, and approximately 40,610 women will die from breast cancer[1]. Although metastatic breast cancer is diagnosed in only 5% of cases at presentation, nearly one third of breast cancer patients with non-metastatic tumors will eventually develop metastases[2].

Breast cancer is considered a genetically heterogeneous and biologically diverse disease. Endocrine therapies that target estrogen receptor (ER) signaling pathways for ER-positive disease and HER2-targeted therapies for HER2-positive disease play a critical role in the treatment of most patients with breast cancer. For patients with ER-positive advanced breast cancer, endocrine therapy is the recommended initial treatment. Since most patients eventually develop resistance to these therapies, the guidelines recommend another endocrine agent when initial therapy fails [3].

Recent advances in elucidating the molecular mechanisms of disease progression have identified the existence of adaptive ‘‘cross-talk” between the ER and various growth factor receptor and intracellular signaling pathways, allowing breast cancer cells to escape the inhibitory effects of endocrine therapy[4]. These findings provide clinical rationale for enhancing or extending endocrine sensitivity by combining endocrine therapy with a targeted agent against compensatory pathways. The PI3K/AKT/mTOR pathway is a crucial mediator of tumor progression [5,6,7]. As the PI3K/Akt/mTOR pathway is heavily deregulated in breast cancer [8,9], inhibitors of mTOR are of interest as potential therapeutic agents for breast cancer patients, with everolimus and temsirolimus being the key drugs considered (S1 Table).

Robust clinical evidence favoring the use of everolimus plus exemestane rather than exemestane alone was demonstrated by the BOLERO-2 trial, a phase 3 study in postmenopausal women with ER-positive, HER2-negative advanced breast cancer progressing or recurring during or following treatment with non-steroidal aromatase inhibitors (AI) (S1 Table). In addition, PFS benefits were consistent across patient subgroups defined by age, race, presence of visceral metastases, and prior chemotherapy [10,11,12].

The greater PFS benefit associated with everolimus plus exemestane was accompanied by increased toxicity, including a higher incidence of mTOR-inhibitor class-effect adverse events (AEs) such as stomatitis, non-infectious pneumonitis (NIP) and hyperglycemia, as well as higher incidences of hematologic toxicity, asthenia, fatigue, and weight loss. Most of these adverse events were mild to moderate in severity, and were generally manageable by dose reduction or interruption. In the BALLET expanded-access study (CRAD001YIC04), the primary reason for discontinuation during the first 3 months of treatment was AEs; for 16% of patients, treatment was discontinued due to toxicity. The most frequent drug-related AEs of grade 3 or more were stomatitis (10%), hyperglycemia (4%), asthenia (6%) and NIP (2%)[13].

A retrospective exploratory analysis of tumor tissue was unable to identify any predictive biomarkers of everolimus efficacy in patient subgroups defined by each of the 4 most frequently altered genes/pathways, when assessed individually (PIK3CA, CCND1, TP53 and FGFR1). Patients with low levels of markers of chromosomal instability had better PFS [14]. No studies to date have evaluated the contribution of germline variants to everolimus treatment outcome in MBC, although some have assessed a limited number of polymorphisms in patients with other types of tumors [15,16].

Thus, there is an urgent need to identify patients that will have early relapse or severe toxicities from everolimus-exemestane, leading to discontinuation of treatment. We carried out an exploratory study with the aiming of defining the impact of germline variation on outcomes following everolimus-exemestane treatment. For this purpose we recruited MBC patients treated with this therapy. We genotyped twelve SNPs in genes involved in everolimus metabolism and transport and genes in the PI3K/AKT/mTOR pathway, and evaluated their association with everolimus pharmacokinetics and toxicity and survival.

## Materials and methods

### Patients

All patients were treated at the 12 de Octubre University Hospital, the hospital institutional ethics committee approved the study in accordance with the principles of Good Clinical Practice, the Declaration of Helsinki, and other applicable local regulations. Written informed consent was obtained from all patients before enrollment.

All patients had locally advanced or MBC and were previously exposed to AI in either the neoadjuvant/adjuvant or palliative setting. Eligible patients were postmenopausal women with ER-positive, HER2–non-amplified, advanced breast cancer whose disease was refractory to previous letrozole, anastrozole or exemestane. Letrozole, anastrozole or exemestane did not have to be the most recent treatment before enrollment, but recurrence or progression during receipt of the most recent systemic therapy had to be documented. Patients who had previously received other anticancer endocrine treatments or prior chemotherapy regimens for advanced disease were included. Patients also had to have an Eastern Cooperative Oncology Group (ECOG) performance status of 2 or less and adequate organ and hematologic functions. Exclusion criteria included previous treatment with mTOR inhibitors.

All patients were treated on an outpatient basis until disease progression or dose-limiting toxicity occurred. All patients had treatment initiated with a daily oral dose of 5 or 10 mg of everolimus and 25 mg of exemestane. Two dose reductions were allowed for grade 3 or higher hematologic or non-hematologic toxicities related to everolimus, or grade 2 for NIP.

### DNA isolation, SNP selection and genotyping

Blood samples for DNA isolation were collected from patients in treatment with exemestane-everolimus in any time of the treatment. Genomic DNA was isolated from peripheral blood using the FlexiGene DNA Kit (Qiagen, Valencia, CA, USA). DNA concentration was quantified by PicoGreen (Invitrogen, Carlsbad, CA, USA).

Twelve SNPs located in genes involved in everolimus metabolism (*CYP3A4*, *CYP3A5* and *CYP2C8*)[17] or everolimus transport (*ABCB1*)[17] or in genes belonging to the PI3K/AKT/mTOR pathway (*FGFR4*, *PHLPP2*, *AKT2*, *PIK3R1*, *RAPTOR* and *AKT1)*[18,19,20]were selected for genotyping taking into consideration the allele frequency and evidence of functionality (Table 1). The latter included published data showing altered activity and/or expression. Reported associations with survival for patients with other tumor types were also considered.

**Table 1:** SNPs included in the study and their genotype frequencies.

Gene	Gene category	SNP	Variant type	MAF	Genotype counts^a^		Reference for selection
***CYP3A4***	Everolimus metabolizing enzyme	rs35599367 C>T	Intronic	0.04	C/C	83 (93%)	[17], [21]
					C/T	6 (7%)	
					T/T	0 (0%)	
***CYP3A5***	Everolimus metabolizing enzyme	rs776746 G>A	Intronic (splicing defect)	0.07	G/G	76 (85%)	[17,22]
					G/A	13 (15%)	
					A/A	0 (0%)	
***CYP2C8***	Everolimus metabolizing enzyme	rs11572080 G>A	Missense (R139K)	0.12	G/G	64 (71%)	[17,23]
					G/A	23 (26%)	
					A/A	3 (3%)	
***ABCB1***	Everolimus transporter	rs1045642 C>T	Synonymous (I1145I)	0.41	C/C	20 (24%)	[17,24,25]
					T/C	48 (56%)	
					T/T	17 (20%)	
***ABCB1***	Everolimus transporter	rs1128503 C>T	Synonymous (G412G)	0.40	C/C	30 (34%	[17,25]
					C/T	39 (45%)	
					T/T	18 (21%)	
***ABCB1***	Everolimus transporter	rs2032582 G>T	Missense (A893S)	0.35	G/G	28 (32%)	[17,25]
					G/T	46 (53%)	
					T/T	13 (15%)	
***FGFR4***	mTOR pathway	rs351855G>A	Missense (G388R)	0.29	G/G	48 (55%)	[15]
					G/A	35 (40%)	
					A/A	5 (6%)	
***PHLPP2***	mTOR pathway	rs61733127 T>C	Missense (L1016S)	0.16	T/T	61 (69%)	[16]
					T/C	25 (28%)	
					C/C	3 (3%)	
***AKT2***	mTOR pathway	rs3730050 G>A	Intronic	0.27	G/G	37 (42%)	[26]
					G/A	43 (48%)	
					A/A	9 (10%)	
***PIK3R1***	mTOR pathway	rs10515074 A>G	Intronic	20	A/A	64 (71%)	[26]
					A/G	24 (27%)	
					G/G	2 (2%)	
***RAPTOR***	mTOR pathway	rs9906827 C>T	Intronic	49	C/C	24 (27%)	[26]
					C/T	44 (50%)	
					T/T	20 (23%)	
***AKT1***	mTOR pathway	rs2494732 A>G	Intronic	43	A/A	30 (34%)	[27]
					A/G	46 (52%)	
					G/G	13 (15%)	

MAF: minor allele frequency in this study.

^a^The number of genotyped patients was 90, but some samples failed genotyping for individual SNPs.

Genotyping was carried out on 15 ng of genomic DNA using the KASPar Technology (KBioscience, UK) and including DNA samples with known genotypes and negative controls. The Sequence Detection System ABI PRISM® 7900HT (Applied Biosystems) was used for the detection of fluorescence and allele assignment. The allele frequencies of the SNPs were similar to those described for Caucasians in1000 Genomes Project and all SNPs had P-values >0.05 for Hardy–Weinberg equilibrium, except *ABCB1* rs2032582 with a minor deviation (P = 0.03). After reviewing the cluster plots, this SNP was included in the analysis.

### Pharmacokinetics

The pharmacokinetic (PK) profile of everolimus was studied by analyzing blood concentration levels in individual samples. Blood samples for pharmacokinetic studies were collected from patients at 1 time point, on day 14 of the first month of treatment. All samples were collected in ethylenediaminetetraacetic acid-containing tubes.

Everolimus concentrations were determined using a chemiluminescentmicroparticle immunoassay (CMIA) by cross-reaction with anti-Sirolimus antibodies, on the Architect i2000SR System (Abbott). Prior to the analysis, a manual pre-treatment step was performed in which the whole blood sample was extracted using a precipitation reagent, then heated (42°C, 10 minutes) and centrifuged (11800 rpm, 4 minutes). The clear supernatant was decanted into a transplant pretreatment tube and analyzed using an Architect i2000SR analyzer. The calibration range of the assay was 0.0–30.0 ng/mL with a sensitivity of 1 ng/mL. The samples that had a concentration greater than 30 ng/ mL were diluted with Calibrator A and retested.

### Outcomes

AEs were recorded from the patients’ medical records retrospectively. Toxicity event was defined as the termination, temporary interruption and/or dose reduction of everolimus. Adverse events were graded using the National Cancer Institute Common Terminology Criteria for Adverse Events, Version 4.03 (NCI CTCAE). The efficacy of everolimus treatment was defined as PFS defined as the time elapsed between treatment initiation by everolimus and tumor progression or death from any cause. Tumor response was determinated at 6 to 12 weeks using Response Evaluation Criteria in Solid Tumors (RECIST) (version 1.1) by each investigators. Patients without documented clinical o radiographic disease progression were censored on the date of the last follow-up. OS was defined as the time elapsed between treatment initiation by everolimus and death from any cause.

The following clinical data were collected from the date of cancer diagnosis to the end of the study: demographic characteristics; number of metastases and their localization; cancer treatment (adjuvant hormonotherapy or chemotherapy; number of hormonotherapy lines in metastatic situation and number of chemotherapy lines in metastatic situation prior to everolimus treatment; everolimus treatment, everolimus initiation, initial dose, date and reason for everolimus termination, temporary(s) interruption(S) and/or dose reduction(S); biological results (hemoglobin, platelets, white cells, neutrophils, albumin, glycaemia, transaminases); presence of AEs.

### Statistical analysis

Associations between SNPs and everolimus concentrations were assessed using Mann-Whitney-U test. Associations with a selection of clinically relevant toxicities (NIP, mucositis, hyperglycemia, leukopenia, lymphopenia and thrombopenia) were assessed using logistic regression. Cox regression was applied to study the associations between SNPs and time to treatment modifications (treatment dose reduction or treatment interruption due to toxicity), PFS and OS.

Factors associated with the outcome variable under study with a P value <0.1 in univariate analyses were included as covariates in multivariable analyses; when no factors reached this threshold, relevant clinical factors were included as covariates, as indicated in the text. An additive (per-allele) genetic model was assessed initially, and for those with P<0.1, alternative genetic models were explored. SPSS v.19 was used for all statistical analyses. P-values less than 0.05 were considered statistically significant.

## Results

### Patient characteristics

A total of 90 women were recruited from October 2011 through January 2015. Baseline characteristics are described in Table 2. For 11 patients the starting dose of everolimus was 5 mg. The median duration of treatment with everolimus was 204 days (range, 13–815 days). Adverse events were consistent with those previously described and are listed in S2 Table.

**Table 2:** Baseline demographic and clinical characteristics.

Characteristic	N (%)*
Median age (range), in years	62 (37–84)
Visceral involvement	55 (61)
Liver	36 (40)
Bone	38 (42)
>3 metastatic sites	37 (41)
ECOG performance status	
0	71 (79)
1	10 (12)
2	9 (10)
Breast cancer IHC	
Estrogen receptor positive	90 (100)
Progesterone receptor positive	75 (84)
Prior hormone therapy in metastatic setting	79 (87)
Median number of lines of therapy (range)	1.5 (0–4)
Anastrozole/letrozole	63 (70)
Fulvestrant	41 (46)
Exemestane	19 (22)
Tamoxifen	23 (26)
Prior chemotherapy in metastatic setting	45 (50)
Median number of lines of therapy (range)	2.4 (0–7)
Taxanes	18 (20)
Antracyclines	17 (19)
Capecitabine	32 (36)

IHC, immunohistochemistry

* Unless otherwise indicated

### Everolimus pharmacokinetics

**Fig 1:**
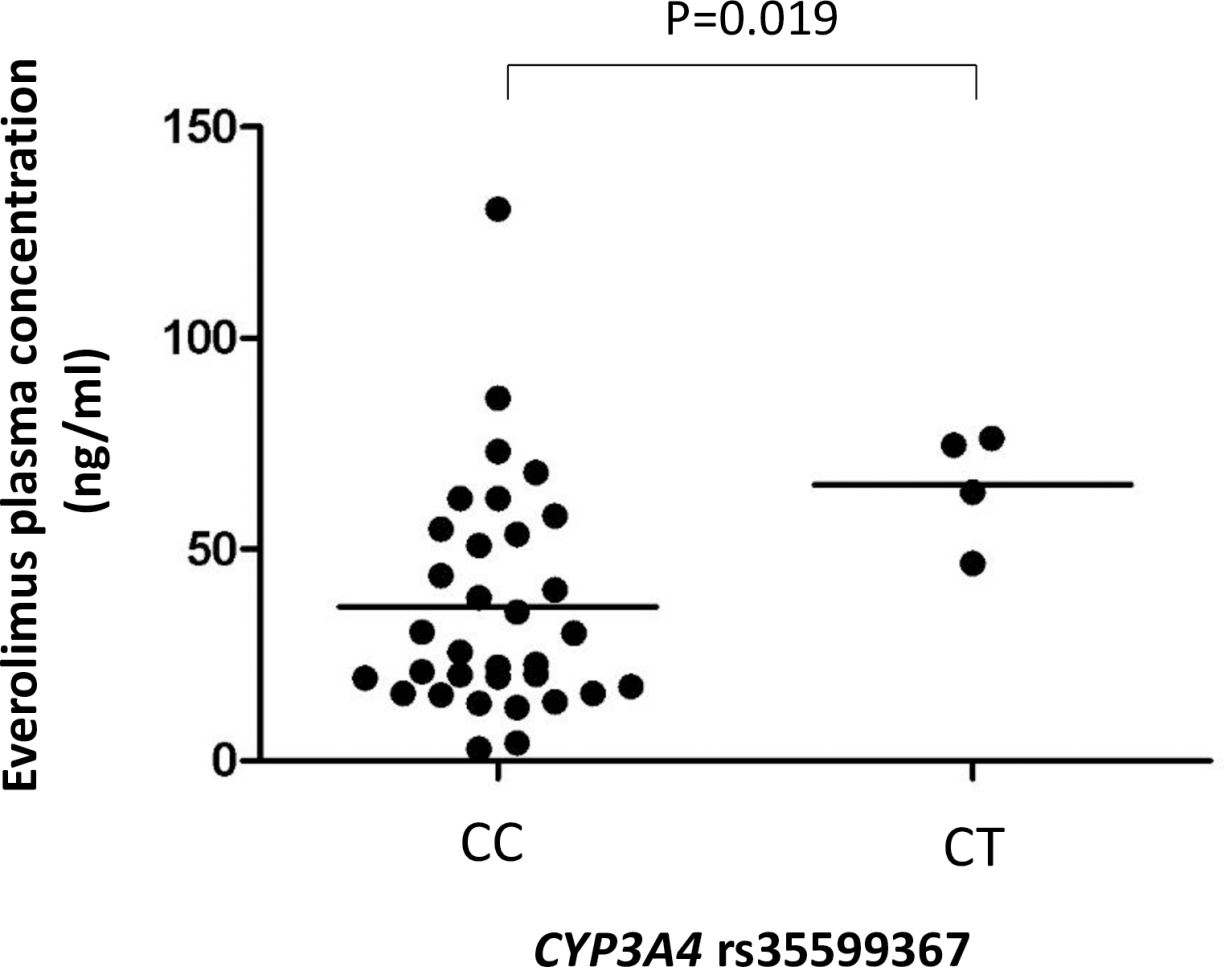
Box plot representing everolimus blood concentration by *CYP3A4* rs35599367 (CYP3A4*22) genotype. “C/C” corresponds to CYP3A4*22 wild type patients (n = 33), and “C/G” to CYP3A4*22 heterozygous carriers (n = 4). Comparison between groups was performed using the Mann-Whitney-U test.

Pharmacokinetic analysis was carried out using data from 37 patients who had PK data on day 14 of the first everolimus cycle. At this time point, the median concentration of everolimus was 30.5 ng/mL (SD = 27.5) with a minimum of 2.8 ng/mL and a maximum of 130.6 ng/mL. Of the 6 SNPs involved in everolimus metabolism (*CYP3A4*, *CYP3A5* and *CYP2C8* genes) and transport (*ABCB1*), only *CYP3A4* rs35599367 (CYP3A4*22 allele) showed a statistically significant association with everolimus concentration (P = 0.019). CYP3A4*22 variant carriers (n = 4) had 2.7-fold higher everolimus concentration compared to wild type patients (median of 69.1ng/mL versus 25.7 ng/mL, respectively; Fig 1). For CYP3A5*1 carriers (n = 4) we found no significantly differences.

### Everolimus toxicities

Of the 90 patients recruited in the study, 69 (76%) experienced at least one adverse event related to everolimus, 43 patients (48%) required a dose reduction to 5 mg daily and 6 (7%) required a second dose reduction to 2.5 mg daily. Eight patients (9%) discontinued treatment due to stomatitis, NIP, fatigue or thrombocytopenia (three, three, three and one patient, respectively). Two patients (2%) died due to AEs, but only one event (NIP) was considered drug-related (S2 Table).

The analysis of time to treatment modification due to toxicity showed a trend (HR = 0.58, 95%CI = 0.33–1.01, P = 0.056) for *FGFR4* rs351855 under an additive genetic model (Table 3). The association under a dominant model was statistically significant for both univariate (HR = 0.50, 95%CI = 0.27–0.93; P = 0.028) and multivariable analyses, the latter correcting for age at diagnosis (HR = 0.52, 95%CI = 0.28–0.97, P = 0.040).

**Table 3:** SNPs associated with toxicity.

Toxicity	Gene	SNP ID	Genetic model	Univariate analysis			Multivariable analysis^a^		
				OR	95% CI	P value	OR	95% CI	P value
Time to treatment modifications due to toxicity	*FGFR4*	rs351855 G>A	Additive	0.58	0.33–1.01	0.056	0.60	0.35–1.06	0.077
			Dominant	0.50	0.27–0.93	0.028	0.52	0.28–0.97	0.040
Leucopenia	*PIK3R1*	rs10515074 A>G	Additive	4.67	1.80–12.1	0.0015	5.03	1.89–13.4	0.0012
Hyperglycemia	*PIK3R1*	rs10515074 A>G	Additive	0.39	0.16–0.95	0.037	0.24	0.07–0.76	0.016
Pneumonitis	*RAPTOR*	rs9906827 C>T	Additive	0.40	0.18–0.91	0.028	0.38	0.16–0.88	0.024
Mucositis	*ABCB1*	rs1045642 C>T	Additive	2.11	1.02–4.37	0.043	2.27	1.08–4.77	0.031
Lymphopenia	*ABCB1*	rs2032582 G>T	Additive	2.33	1.12–4.81	0.023	2.23	1.07–4.67	0.033

^a^The multivariable analysis performed for time to treatment modifications, mucositis, pneumonitis, hyperglycemia, leucopenia and lymphopenia included the following covariates: age, presence of visceral disease, previous pneumonitis events, diabetes mellitus status, number of previous chemotherapy lines and number of previous chemotherapy lines, respectively.

Associations between SNPs and clinically relevant everolimus toxicities were detected using logistic regression analysis (Table 3). For mucositis, patients with the T-allele of *ABCB1* rs1045642 had higher risk of toxicity (OR = 2.30, 95%CI = 1.08–4.77, P = 0.031; multivariable analysis). The A-allele of *RAPTOR* rs9906827 was associated with lower risk of non-infectious pneumonitis (OR = 0.38, 95%CI = 0.16–0.88, P = 0.024; multivariable analysis). The minor allele of *PIK3R1* rs10515074 was associated with reduced risk of hyperglycemia (OR = 0.24, 95%CI = 0.07–0.76, P = 0.016), but increased risk of leucopenia (OR = 5.03, 95%CI = 1.89–13.35, P = 0.001); there was also a trend with lymphopenia (OR = 2.48, 95%CI = 0.92–6.69, P = 0.073), all in multivariable analysis. *ABCB1* rs2032582 was significantly associated with lymphopenia risk (OR = 2.23, 95%CI = 1.07–4.67, P = 0.033; multivariable analysis). No SNPs were associated with thrombocytopenia.

### Progression-free survival and overall survival in metastatic breast cancer

**Fig 2:**
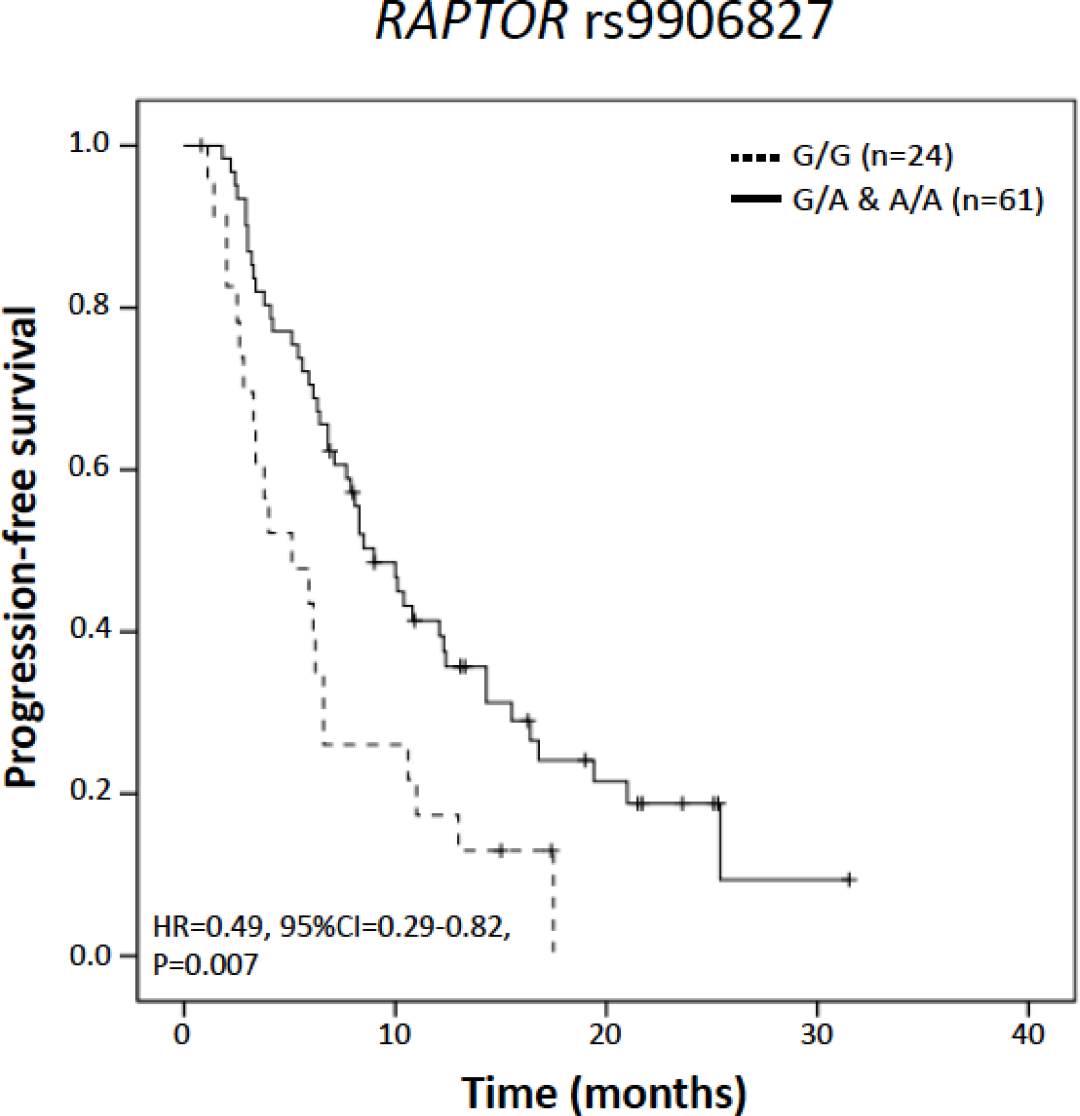
Kaplan-Meier curve for progression free survival by RAPTOR rs9906827 genotype. P-value corresponds to Cox regression analysis under a dominant genetic model including the number of previous chemotherapy lines as covariate. HR, hazard ratio; CI, confidence interval.

The minor allele of *RAPTOR* rs9906827 was associated with longer PFS in univariate Cox regression analysis both in an additive and dominant genetic model (HR = 0.65, 95%CI = 0.45–0.94, P = 0.023 and HR = 0.49, 95%CI = 0.29–0.82, P = 0.007, respectively; Fig 2). After the inclusion of age, number of previous chemotherapy lines (Fig 2), number of previous lines of hormone-therapy and presence of previous visceral disease, the association remained statistically significant with no substantial changes in estimated HRs or P-values. No SNPs were significantly associated with OS.

## Discussion

The BOLERO-2 trial demonstrated a clinically meaningful improvement in PFS with everolimus plus exemestane therapy in patients with ER–positive/HER2-negative advanced breast cancer [10,11].Despite these encouraging results, not all patients benefit from everolimus, and an optimal target patient population for this drug is yet to be defined. Moreover, the benefits of treatment with everolimus come with an increased incidence of mTOR inhibitor-associated toxicities. Thus, there is an urgent need for biomarkers that can predict response to everolimus and better define the ideal target patient group.

Germline variants have previously been shown to be accurate biomarkers of drug toxicities, and their stability over a subject´s life and easy assessment make them especially attractive. The aim of this study was to explore whether germline variants are implicated in everolimus toxicity and response to treatment. We performed a pharmacogenetic analysis using 90 MBC patients treated with exemestane-everolimus, including a subset in which their PK profile was determined. To the best of our knowledge, this is the first study investigating germline predictors of exemestane-everolimus outcome in MBC. The impact on everolimus activity of SNPs located in PI3K/AKT/mTOR pathway genes and the alteration of everolimus plasma concentration caused by theCYP3A4*22variant, point towards germline variation as a relevant factor influencing everolimus outcomes in MBC. These results warrant replication in prospective patient series.

Everolimus is used in transplant recipients, tuberous sclerosis complex and metastatic breast, kidney and neuroendocrine pancreas cancer. The transplantation literature includes numerous associations between polymorphisms in genes encoding drug metabolizing enzymes and drug transporters and the pharmacokinetics of immunosuppressive drugs (e.g. tacrolimus, cyclosporine, everolimus). Most of these studies have been retrospective in design and the most relevant associations correspond to variants in *CYP3A5* and *ABCB1*[28,29], [30,31], however, most studies of everolimus pharmacokinetics have given negative results[32,33,34,35]. In this study, we found that CYP3A4*22 resulted in significantly higher plasma levels of everolimus, consistent with the decreased activity caused by the CYP3A4*22 allele [21], while no effect was detected for CYP3A5*3 with the same number of carrier patients This suggests a greater influence of CYP3A4*22 than CYP3A5*3 in everolimus pharmacokinetics, at least in MBC patients. Furthermore, everolimus dosing in cancer is much higher than in transplant patients (i.e. 5–10 mg/day versus 2-5mg/day), thus, it is conceivable that in the cancer setting, CYP3A4*22 might exert a greater and more clinical relevant effect on drug toxicity than in the transplant setting. In this study we did not find statistically significant differences in the toxicities evaluated for CYP3A4*22 variant carriers, however, only 6 carriers were identified, giving low statistical power. Thus, further studies are needed to clarify the impact of CYP3A4*22 on everolimus toxicity.

Class-effect toxicities during mTOR inhibitor therapy are well characterized. The most common adverse events observed in everolimus clinical trials include stomatitis (50%), rash (40%), immunosuppression (40%), NIP (15%) and hyperglycemia (15%) [10,36,37,38,39]. Treatment modifications guided by biomarkers predictive of toxicity could minimize severe toxicities and increase optimal outcomes. SNP rs10515074 in *PIK3R1*, a gene encoding the 85 kD regulatory subunit of phosphatidylinositol 3-kinase enzyme, which is an upstream member that triggers thePI3K/AKT/mTOR signaling pathway, was associated with hyperglycemia and leucopenia. This variant has previously been associated with survival in muscle invasive and metastatic bladder cancer patients [40]. Furthermore, RAPTOR (regulatory associated protein of mTOR) has a positive role in nutrient signaling and in the control of cell size[41], and its intronic variant rs9906827 has been found to be associated with survival in muscle invasive and metastatic bladder cancer patients [40]. In our study in breast cancer patients treated with exemestane and everolimus, a statistically significant association in the same direction (variant allele associated with better outcome) was observed for *RAPTOR* rs9906827 and PFS. Thus, our results suggests that constitutive variation in the PI3K/AKT/mTOR pathway could result in alteration in susceptibility to toxicities caused by drugs inhibiting this pathway and also exert an effect in tumor outcome during treatment. In addition, SNPs in *ABCB1* were associated with increased risk of mucositis (rs1045642) and lymphopenia (rs2032582). These associations remained significant after multivariable analysis, and warrant validation in an independent series.

Key limitations affecting our study were the limited sample size and the lack of replication in an independent prospective study. However, this is a first exploratory study aimed at generating hypothesis, and with the exception of CYP3A4*22 allele, the SNPs associated with everolimus outcome have a relative high allele frequency, increasing the power of the study. Another limitation was that schedule and dose modifications were not dictated by protocol (they reflect real life drug management), and the timing for radiological assessments was determined by individual clinicians. Thus, courses of treatment were not standardized and outcomes were assessed with regard to clinical practice.

In conclusion, this is the first study exploring the impact of germline variation on exemestane-everolimus outcome in MBC. Our results provide evidence that the CYP3A4*22variant influences everolimus PK and suggest that polymorphisms in *ABCB1* and PI3K/AKT/mTOR pathway genes could influence everolimus toxicity and response in MBC. These results require replication in an independent prospective series. If confirmed, these genetic variants could be used to inform individualized metastatic breast cancer treatment.

## Supporting information

S1 Table Summary of published randomized clinical studies evaluating the efficacy of combination of mTOR-inhibitor and hormonotherapy in patients with hormone receptor positive advanced breast cancer. (DOCX)

S2 Table Adverse events related to everolimus. (DOCX)
