# Supplementary material for: Polymorphisms associated with everolimus pharmacokinetics, toxicity and survival in metastatic breast cancer
Source: PLoS One. 2017 Jul 20;12(7):e0180192. doi: 10.1371/journal.pone.0180192 (PMC5519037; doi:10.1371/journal.pone.0180192)
Supplement: S1 Table — (DOCX) [file pone.0180192.s001.docx]

**Supplementary Table 1.** Summary of published randomized clinical studies evaluating the efficacy of combination of mTOR-inhibitor and hormonotherapy in patients with hormone receptor positive advanced breast cancer.

| **Trial ID** | **Treatment arms** | **Study size, N** | **Setting** | **Clinical outcomes** | **Reference** |
| --- | --- | --- | --- | --- | --- |
| TAMRAD NCT01298713 | EVE + TAM versus TAM | 111 | First-line/second-line. AI-resistant metastatic (after prior AI treatment) | PFS  EVE + TAM = 8.6 months  TAM = 4.5 months  (HR, 0.54; 95% CI, 0.36 to 0.81; p = 0.0021) | [1] |
| Baselga et al 2005  NCT00062751 | TEM 10 mg +LET versus TEM 30mg + LET versus LET | 92 | First-line | PFS  LET + PBO = 11.6 months  TEM 10 + LET = 13.2 months  TEM 30 + LET = 11.5 months | [2] |
| HORIZON  NCT00083993 | TEM + LET versus PBO + LET | 1112 | First-line | PFS  TEM + LET = 8.9 months  PBO + LET = 9.0 months  (HR 0.90; 95% CI 0.76 to 1.07; p = 0.25) | [3] |
| BOLERO-2  NCT00863655 | EVE + EXE versus PBO + EXE | 724 | First-line/second-line. Metastatic, refractory to previous letrozole or anastrozole | PFS  EXE+EVE = 11 months  EXE+PBO = 4,1 months  (HR, 0.38; 95% CI, 0.31 to 0.48; p < 0.001) | [4, 5] |
| Bhattacharyya et al 2011 | TAM + SIR versus TAM | 200 | Metastatic patients, who could not afford AI | Percentage responding to treatment 36% vs 68% (average ER status 4 to 8, median = 6) and time to progression − 9 months vs 16 months | [6] |
| Bhattacharyya et al 2011 | TAM + SIR versus TAM | 200 | Metastatic patients, who had failed AI and/or tamoxifen | Percentage responding to treatment of 4% vs 40% and time to progression − 3 months vs 11 months | [6] |
| PrECOG 0102  NCT01797120 | PBO + FVT versus FVT + EVE | 131 | First-line/second-line. Metastatic, refractory to previous letrozole or anastrozole | PFS  FVT+EVE = 10.4 months  EXE+PBO = 5,1 months  (HR, 0.61; 95% CI, 0.40 to 0.92; p = 0.02) | [7] |

N, number of patients; EVE, everolimus; TAM, tamoxifem; TEM, temsirolimus; PBO, placebo; EXE, exemestane; LET, letrozole; SIR, sirolimus; ER, estrogen receptor; PFS, progression-free survival; HR, hazard ratio; CI, confidence interval; AI, aromatase inhibitor.

1. Bachelot T, Bourgier C, Cropet C, Ray-Coquard I, Ferrero J-M, Freyer G, et al. Randomized phase II trial of everolimus in combination with tamoxifen in patients with hormone receptor–positive, human epidermal growth factor receptor 2–negative metastatic breast cancer with prior exposure to aromatase inhibitors: A GINECO study. Journal of Clinical Oncology. 2012;30(22):2718-24.

2. Baselga J, Roche H, Fumoleau P, Campone M, Colomer R, Cortes-Funes H, et al. Treatment of postmenopausal women with locally advanced or metastatic breast cancer with letrozole alone or in combination with temsirolimus: a randomized, 3-arm, phase 2 study. Breast Cancer Research and Treatment. 2005;94:S62.

3. Wolff AC, Lazar AA, Bondarenko I, Garin AM, Brincat S, Chow L, et al. Randomized phase III placebo-controlled trial of letrozole plus oral temsirolimus as first-line endocrine therapy in postmenopausal women with locally advanced or metastatic breast cancer. Journal of Clinical Oncology. 2012;31(2):195-202.

4. Piccart M, Hortobagyi GN, Campone M, Pritchard KI, Lebrun F, Ito Y, et al. Everolimus plus exemestane for hormone-receptor-positive, human epidermal growth factor receptor-2-negative advanced breast cancer: overall survival results from BOLERO-2dagger. Ann Oncol. 2014;25(12):2357-62. doi: 10.1093/annonc/mdu456. PubMed PMID: 25231953.

5. Baselga J, Campone M, Piccart M, Burris HA, 3rd, Rugo HS, Sahmoud T, et al. Everolimus in postmenopausal hormone-receptor-positive advanced breast cancer. N Engl J Med. 2012;366(6):520-9. doi: 10.1056/NEJMoa1109653. PubMed PMID: 22149876.

6. Bhattacharvva G, Biswas J, Singh J, Singh M, Govindbabu K, Ranade A, et al. Reversal of tamoxifen resistance (hormone resistance) by addition of sirolimus (mTOR inhibitor) in metastatic breast cancer. European Journal of Cancer. 2011;47:9.

7. Kornblum N, Manola J, Klein P, Ramaswamy B, Brufsky A, Stella P, et al. Abstract S1-02: PrECOG 0102: A randomized, double-blind, phase II trial of fulvestrant plus everolimus or placebo in post-menopausal women with hormone receptor (HR)-positive, HER2-negative metastatic breast cancer (MBC) resistant to aromatase inhibitor (AI) therapy. AACR; 2017.
