# Supplementary material for: Polymorphisms associated with everolimus pharmacokinetics, toxicity and survival in metastatic breast cancer
Source: PLoS One. 2017 Jul 20;12(7):e0180192. doi: 10.1371/journal.pone.0180192 (PMC5519037; doi:10.1371/journal.pone.0180192)
Supplement: S2 Table — (DOCX) [file pone.0180192.s002.docx]

**Supplementary Table 2.** Adverse events related to everolimus^a^**.**

| **Adverse event** | **Any grade** | | **Grade 3-5** | | **Dose reduction/ suspension** | |
| --- | --- | --- | --- | --- | --- | --- |
|  | **N** | **%** | **N** | **%** | **N** | **%** |
| Anemia | 70 | 78 | 7 | 8 | 0 | 0 |
| Lymphopenia | 68 | 76 | 11 | 12 | 3 | 3 |
| Mucositis | 61 | 68 | 6 | 7 | 14 | 16 |
| Skin Rash | 46 | 51 | 1 | 1 | 6 | 7 |
| Hyperglycemia | 45 | 50 | 14 | 16 | 2 | 2 |
| Hypercholesterolemia | 40 | 44 | 1 | 1 | 0 | 0 |
| Leucopenia | 39 | 43 | 3 | 3 | 3 | 3 |
| Thrombopenia | 36 | 40 | 2 | 2 | 2 | 2 |
| GOT/GPT increase | 33 | 37 | 3 | 3 | 5 | 6 |
| Diarrhea | 27 | 30 | 5 | 6 | 8 | 9 |
| Pneumonitis | 21 | 23 | 3 | 3 | 11 | 12 |
| Peripheral edema | 20 | 22 | 0 | 0 | 3 | 3 |

GOT, Glutamyl oxaloacetic transaminase; GPT, Glutamyl pyruvic transaminase

**^a^** Adverse events were graded according to the Common Toxicity Terminology Criteria for Adverse Events (CTCAE) (version 4.03)
